# Effect of traditional Chinese medicine (TCM) on survival, quality of life, and immune function in patients with ovarian carcinoma: A protocol for systematic review and meta analysis

**DOI:** 10.1097/MD.0000000000023904

**Published:** 2021-01-15

**Authors:** Shuxia Ge, Qianqian Xing, Anqi Zhang, Yucui Wang

**Affiliations:** a Department of Obstetrics and Gynecology, Liaocheng People's Hospital; b Department of Quality Control, Liaocheng City Dongchangfu District Maternal and Child Health Hospital; c Department of Central Laboratory, Liaocheng People's Hospital, Liaocheng 252000, Shandong Province, P.R. China.

**Keywords:** traditional Chinese medicine, ovarian carcinoma, meta-analysis, immune function, efficacy

## Abstract

**Background:**

Traditional Chinese medicine (TCM) has been widely applied as promising adjunctive drugs for ovarian carcinoma (OC) in China and other Asian countries. However, its exact clinical efficacy and safety is still not well investigated. In this study, we aimed to summarize the efficacy of TCM on survival, quality of life (QoL), and immune function in patients with OC through the meta-analysis.

**Methods:**

Relevant clinical trials of TCM for the treatment OC patients will be searched in Cochrane Library, Web of Science, Google Scholar, PubMed, Medline, Embase, China Scientific Journal Database, China National Knowledge Infrastructure, Chinese Biomedical Literature Database, and Wanfang Database from their inception to November 2020. Two researchers will perform data extraction and risk of bias assessment independently. The clinical outcomes, including overall survival (OS), QoL, immune function, tumor markers, and adverse events, were systematically evaluated by using Review Manager 5.3 and Stata 14.0 statistical software.

**Results:**

The results of this study will provide high-quality evidence for the effect of TCM on survival, QoL and immune function in patients with OC.

**Conclusion:**

The conclusions of this meta-analysis will be published in a peer-reviewed journal, and draw an objective conclusion of the efficacy of TCM on survival, QoL, and immune function in patients with OC.

**Trial registration number:**

INPLASY2020110104.

## 1 Introduction

Ovarian carcinoma (OC), the second most common cause of gynecologic tumor death in women, accounts for about 20% of all female reproductive cancers around the world.^[1,2]^ There are 184,799 deaths occurred in 2018 according to global cancer statistics.^[3,4]^ OC can occur at any age, more common in patients older than 50 years.^[1,2]^ With increasing life-expectancy, the number of OC cases diagnosed each year is increasing significantly. Despite the improvement of diagnostic and therapeutic methods in the past decades, the prognosis of OC remains unsatisfactory.^[5,6]^ Over 60% to 70% of OC patients are diagnosed at advanced stage, with 5-year survival rates less than 45%.^[1,2,5,6]^ Conventional treatment options for OC include surgery, radiotherapy, chemotherapy, or a combination of them, depending on the severity of the disease.^[5,6]^ However, their clinical applications are limited by failing to thoroughly eliminate tumor cells, drug resistance, and other adverse effects.^[7–9]^ In view of these drawbacks to conventional therapy, there is a growing interest in the development of a new regimen with better tolerance and lower toxicity for patients with OC.

Traditional Chinese medicine (TCM), as an essential component of complementary and alternative medicine, has gained more and more attention for malignant tumors.^[10–18]^ TCMs are prepared by extracting and purifying the effective and active compounds from herbs, insects, or animals via modern scientific techniques and methods.^[16]^ The anticancer TCMs are mainly used for adjuvant radiotherapy and chemotherapy against tumors by reducing toxicity, enhancing efficiency, ameliorating symptoms, and improving the immune status in clinical use.^[16]^ Several studies have indicated that the combination of TCM and classic radiochemotherapy not only exerts an enhanced therapeutic effect against OC, but also improves the quality of life (QoL) and immune function of patients.^[17–21]^ Despite the intensive clinical studies, its clinical efficacy was still not well investigated. In this study, we are prepared to summarize the efficacy of TCM on survival, QoL, and immune function in patients with advanced OC through the meta-analysis, in order to provide a helpful evidence for clinicians to formulate the best postoperative adjuvant treatment strategy for OC patients.

### 1.1 Review question

Is TCM effective on survival, QoL, and immune function in patients with OC?

### 1.2 Objective

A systematic review and meta-analysis will be performed to systematically evaluate the efficacy of TCM on survival, QoL, immune function, tumor markers, and adverse effect in patients with OC.

## 2 Methods

### 2.1 Study registration

This protocol of systematic review and meta-analysis has been registered on the International Platform of Registered Systematic Review and Meta-Analysis Protocols (INPLASY). The registration number was INPLASY2020110104 (URL: https://inplasy.com/inplasy-2020-11-0104/). It will be reported according to Preferred Reporting Items for Systematic Review and Meta-Analysis Protocols (PRISMA-P) guidelines.^[22]^

### 2.2 Ethics

As the program does not require the recruitment of patients and the collection of personal information, no further ethical approval is required.

### 2.3 Eligibility criteria

#### 2.3.1 Types of studies

Randomized controlled trials (RCTs) or prospective controlled clinical trials that investigated the efficacy of TCM on survival, QoL, tumor markers, and immune function in patients diagnosed with OC will be included in this systematic review. There will be no restrictions for blinding, population characteristics, and duration of trials.

#### 2.3.2 Type of participants

Patients with histologically proved OC were included in this study. No restrictions regarding age, gender, racial, region, education, and economic status were reported. Patients with other malignancies are not included.

#### 2.3.3 Types of interventions

In the experimental group, OC patients must be treated with TCM alone or in combination with other conventional treatment methods, including surgery, radiotherapy, chemotherapy, and so on. TCM involving extracts from herbs or insects or animals, single or mixture formulas regardless of their compositions or forms. There will be no restrictions with respect to dosage, duration, frequency, or follow-up time of treatment.

#### 2.3.4 Comparator

There will be no restrictions with respect to the type of comparator. The comparators are likely to include surgery, radiotherapy, chemotherapy, supportive care, and other therapeutic methods.

#### 2.3.5 Type of outcome measurements

##### 2.3.5.1 Primary outcomes

- Overall survival (OS), the time from the date of randomization to death from any cause;
- QoL obtained from the corresponding scale;
- Immune function indicators: CD3^+^, CD4^+^, CD8^+^, NK cells percentage, CD4^+^/CD8^+^ cell ratios, and serum cytokines level (IL-2, IL-4, IFN-γ, TNF-α, and so on).

##### 2.3.5.2 Secondary outcomes

- Overall response rate (ORR) and disease control rate (DCR);
- Tumor markers: HE4, CA125, CEA, and CA199;
- Adverse effects: Gastrointestinal adverse reactions, leukopenia, hepatorenal toxicity, and so on.

#### 2.3.6 Exclusion criteria

Duplicated studies, papers without sufficient available data, noncomparative clinical trials, literature reviews, meta-analysis, meeting abstracts, case reports and series, and other unrelated studies will be excluded from analysis.

### 2.4 Information sources

Relevant clinical trials of TCM for the treatment OC patients will be searched in Cochrane Library, Web of Science, Google Scholar, PubMed, Medline, Embase, China Scientific Journal Database, China National Knowledge Infrastructure, Chinese Biomedical Literature Database, and Wanfang Database from their inception to November 2020. Language is limited to English and Chinese.

### 2.5 Search strategy

Experienced systematic review investigators will be invited to develop a search strategy, in order to perform a comprehensive search. The search terms include “ovarian cancer” or “ovarian oncology” or “ovarian tumor” or “ovarian carcinoma” or “epithelial ovarian carcinoma” or “oophoroma” or “luan cao ai” or “luan cao zhong liu” or “OC” or “EOC” and “traditional Chinese medicine” or “traditional Chinese drug” or “Chinese herbal preparation” or “traditional Chinese preparation” or “Chinese materia medica preparation” or “Chinese patent medicine” or “Chinese herbal injection” or “Traditional Chinese medicine injection” or “zhongyao” or “TCM,” and so on. The preliminary retrieval strategy for PubMed is provided in Table 1, which will be adjusted in accordance with specific databases.

**Table 1:** Searching strategy in PubMed.

Search strategy
#1. “Ovarian cancer” or “Ovarian tumor” or “Ovarian neoplasm” or “Ovarian carcinoma” or “Ovarian malignant” or “Ovarian oncology” or “Epithelial ovarian cancer” or “Epithelial ovarian tumor” or “Epithelial ovarian neoplasm” or “Epithelial ovarian carcinoma” or “Epithelial ovarian malignant” or “Epithelial ovarian oncology” or “oophoroma” or “Cancer of the ovarian” or “Cancer of the epithelial ovarian” or “OC” or “EOC” [Title/Abstract].
#2. “Ovarian cancer” [MeSH].
#3. #1 or #2.
#4. “Chinese medicine” or “traditional Chinese medicine” or “traditional Chinese drug” or “Chinese herbals” or “Chinese herbal injection” or “Traditional Chinese medicine injection” or “Chinese herbal preparation” or “traditional Chinese preparation” or “Chinese materia medica preparation” or Chinese patent medicine” or “TCM” [Title/Abstract].
#5. “Chinese medicine” or [MeSH].
#6. #4 or #5
#7. #3 and #6
#8. Limit #7 to “human”
#9. Limit #8 to “Clinical trial” [Publication Type]
#10. Limit #9 to yr = “-November 2020”

### 2.6 Study selection and data extraction

#### 2.6.1 Study selection and management

**Figure 1:**
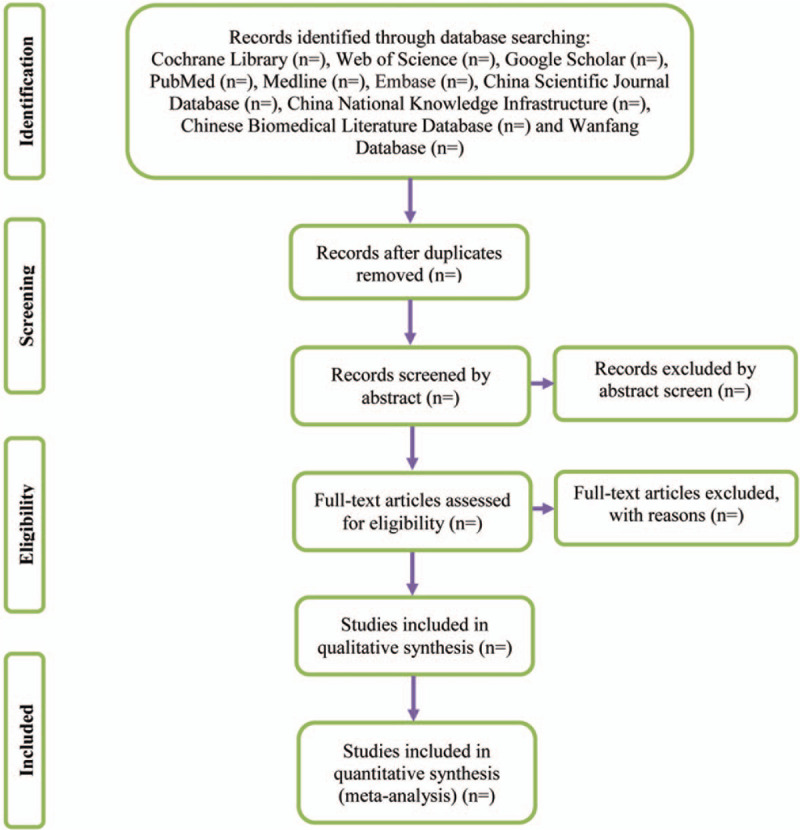
Study selection process for the meta-analysis.

Two experienced authors (Shuxia Ge and Qianqian Xing) will be reviewed independently to identify potential trials by assessing the titles and abstracts. The full text will be further reviewed to determine potential eligible studies. A PRISMA-compliant flow chart (Fig. 1) will be used to describe the selection process of eligible trials. Excluded studies and reasons for exclusion will be recorded. Endnote X7 software will be used for literature managing and records searching. Disagreements between the 2 researchers will be resolved by consensus or by a third independent investigator (Anqi Zhang).

#### 2.6.2 Data extraction and management

After screening the text, the 2 investigators (Shuxia Ge and Qianqian Xing) will independently extract the information contained in the eligible literature. The extracted data are as follows:

Study characteristics and methodology: country of study, the first author's name, year of publication, randomization, sample size, periods of data collection, follow-up duration, outcome measures, inclusion and exclusion criteria, and so on.

Participant characteristics: age, gender, tumor stage, tumor size, diagnostic criteria, and so on.

Interventions: therapeutic means, types of TCM, dose, administration route, course of treatment, and duration of treatment, and so on.

Outcome and other data: ORR, DCR, OS, QoL, immune indexes [(CD3^+^, CD4^+^, CD8^+^, NK cells percentage, and CD4^+^/CD8^+^ cell ratios), tumor markers (HE4, CA125, CEA and CA199) and serum cytokines level (IL-2, IL-4, IFN-γ, and TNF-α)], and adverse effects, and so on.

When any data are missing or insufficient, we will contact original authors by using email. If those relevant data are not acquired, we will only analyze the available data, and discuss its impact as a limitation.

### 2.7 Quality assessment

Two researchers (Shuxia Ge and Qianqian Xing) will independently assess the quality of the included RCTs in accordance with the Cochrane Handbook of Systematic Reviewers. This assessment tool includes 7 items: random sequence generation, allocation concealment, blinding of participants and personnel, blinding of outcome assessment, incomplete outcome data, selective reporting, and other bias.^[23,24]^ Each item will be evaluated at 3 levels: low risk, unclear, and high risk. Effective Practice and Organisation of Care (EPOC) guidelines will be used to assess the risks of non-RCTs.^[25]^ Any disagreements will be resolved via discussion with a third researcher (Anqi Zhang).

### 2.8 Data synthesis

Review Manager 5.3 (Nordic Cochran Centre, Copenhagen, Denmark) and Stata 14.0 (Stata Corp., College Station, TX) statistical software will be used to pool the data and carry out the data analysis. Continuous data will be presented as mean difference (MD) or standardized mean difference (SMD) with their 95% confidence intervals (95% CIs). Dichotomous data will be recorded as risk ratio (RR) with 95% CIs. A 2-tailed *P* < .05 was considered statistically significant.

### 2.9 Assessment of heterogeneity

Heterogeneity of treatment effects across trials was assessed by χ^2^ statistics and the *I*^2^ statistics.^[26]^ When the *P* value was >.1, and *I*^*2*^ was < 50%, it was suggested that there was no statistical heterogeneity and the Mantel--Haenszel fixed-effects model was used for meta-analysis. Otherwise, a random-effects mode will be used to calculate the outcomes.

### 2.10 Subgroup and meta-regression analysis

We will explore sources of heterogeneity with respect to age, tumor stage, region, and types of TCM by subgroup analysis and meta-regression when the *P* value was <.1, and *I*^2^ was >50%.

### 2.11 Sensitivity analysis

Sensitivity analysis will be conducted to assess the reliability and robustness of the aggregation results via eliminating trials with low quality. A summary table will report the results of the sensitivity analyses.

### 2.12 Publication bias

Funnel plot will be performed to analyze the existence of publication bias if 10 or more literatures are included in this meta-analysis. If the funnel diagram has poor symmetry, it indicates publication bias.^[27]^ Begg and Egger regression test are used to further verify the existence of publication bias.^[28,29]^ If publication bias existed, a trim-and-fill method should be applied to adjust the pooled OR.^[30]^

### 2.13 Assess the quality of evidence

The guidelines of the Grading of Recommendations, Assessment, Development, and Evaluation (GRADE) will be used to assess the quality of evidence and the strength of the main result recommendations.^[31]^ The quality of all evidence will be assessed at 4 levels: high, moderate, low, and very low.

### 2.14 Dissemination

The results of this study will be published in a peer-reviewed journal, and provide reliable evidence for clinicians to formulate the best postoperative adjuvant treatment strategy for OC patients.

## 3 Discussion

The chemoradiotherapy regimens commonly used to treat OC often cause serious adverse effects, which severely affect the QoL and immune function of patients.^[17–21]^ Therefore, seeking an alternative therapy that can improve the QoL and immune function of patients is urgently required for tumor treatment. Several studies have recognized that TCM have a unique advantage in the treatment of malignant tumors by inhibiting the growth of cancer cells, enhancing immunity of human body, decreasing cancer relapses and metastases, and mitigating the progress of the disease.^[17–21,32–38]^ Piao et al^[21]^ found that complementary treatment with mistletoe extracts can beneficially reduce the side effects of chemotherapy and thus improve QoL of patients with breast, ovarian and nonsmall cell lung cancer. Research by Chan et al^[18]^ indicated that TCM did not improve QoL of cancer patients but did have some effects in terms of maintaining immune function. The results of study by Wang et al^[32]^ showed that the clinical application of Chinese herbal medicine not only significantly enhanced the curative effects of conventional treatment but also effectively improved QoL and clinical symptoms in patients with OC. Tao et al^[33]^ found that the compound fuling granule (CFG, a traditional Chinese drug) can significantly suppresses OC cell proliferation by cell cycle arrest, apoptosis, and senescence. In addition, CFG can also inhibit the invasion and migration ability of OC cells induced by TGF-β. All these findings indicate that TCM may use as a promising adjuvant therapeutic method for the treatment of OC. Although there was statistical analysis of published clinical trials, the exact effects of TCM on survival, QoL, and immune function in patients with OC were still not systematically investigated. This meta-analysis will conduct a systematic, comprehensive, and objective evaluation of TCM-based adjuvant therapy. There are some limitations that may affect the drawn conclusion. Due to the different TCM drugs, tumor stage and duration of treatment among included trials that may cause a certain degree of heterogeneity. In addition, there may be a language bias with the limitation of English and Chinese studies. All in all, we hope the findings of this analysis will provide a helpful evidence for clinicians to formulate the best postoperative adjuvant treatment strategy for patients with advanced OC, and also provide scientific clues for researchers in this field.

## Author contributions

**Conceptualization:** Yucui Wang and Shuxia Ge.

**Data curation:** Shuxia Ge and Qianqian Xing.

**Formal analysis:** Shuxia Ge and Qianqian Xing.

**Funding acquisition:** Anqi Zhang.

**Investigation:** Shuxia Ge and Qianqian Xing.

**Methodology:** Shuxia Ge, Qianqian Xing and Anqi Zhang.

**Project administration:** Yucui Wang.

**Resources:** Yucui Wang and Shuxia Ge.

**Software:** Yucui Wang and Shuxia Ge.

**Supervision:** Yucui Wang and Shuxia Ge.

**Validation:** Yucui Wang and Anqi Zhang.

**Visualization:** Shuxia Ge and Qianqian Xing.

**Writing – original draft:** Shuxia Ge and Qianqian Xing.

**Writing – review & editing:** Yucui Wang and Anqi Zhang.

## Abbreviations

Abbreviations: CIs = confidence intervals, DCR = disease control rate, GRADE = Grading of Recommendations Assessment, Development, and Evaluation, INPLASY = International Platform of Registered Systematic Review and Meta-Analysis Protocols, MD = Mean difference, OC = ovarian carcinoma, ORR = overall response rate, OS = overall survival, PRISMA-P = Preferred Reporting Items for Systematic Reviews and Meta-Analyses Protocols, QoL = quality of life, RCTs = randomized controlled trials, RR = risk ratio, SMD = standardized mean difference, TCM = Traditional Chinese medicine.
